# The Influence of Food Density, Flock Size, and Disturbance on the Functional Response of Bewick’s Swans (*Cygnus columbianus bewickii*) in Wintering Habitats

**DOI:** 10.3390/ani9110946

**Published:** 2019-11-10

**Authors:** Chao Yu, Lizhi Zhou, Nazia Mahtab, Shaojun Fan, Yunwei Song

**Affiliations:** 1 School of Resources and Environmental Engineering, Anhui University, 111 Jiulong Road, Hefei 230601, China; yc-107@163.com (C.Y.);; 2 Anhui Province Key Laboratory of Wetland Ecosystem Protection and Restoration (Anhui University), 111 Jiulong Road, Hefei 230601, China; 3 Department of Resources Conservation and Utilization, Anhui Shengjin Lake National Nature Reserve, Dongzhi 247200, China

**Keywords:** functional response, feeding rate, handling time, searching rate, vigilance, food item density, flock size, Bewick’s swans

## Abstract

**Simple Summary:**

Changes in environmental conditions cause animals to adjust their behavioral strategies to survive. We investigated foraging behavior in different habitats of wintering Bewick’s swans. We found that the observed feeding rate was not affected by food density but showed a negative relationship with flock size and disturbance time. Handling time had a negative relationship with food density and flock size, but a positive relationship with disturbance. Searching rate was negatively correlated with food density, flock size, and disturbance time. This provides insight into how wintering waterbirds adapt their foraging behavior in complex environments.

**Abstract:**

Perceiving how animals adjust their feeding rate under a variety of environmental conditions and understanding the tradeoffs in their foraging strategies are necessary for conservation. The Holling functional response, which describes the relationship of feeding rate and food density to searching rate and handling time, has been applied to a range of waterbirds, especially with regard to Type II functional responses that describe an increasing feeding rate with food density but at a decelerating rate as the curve approaches the asymptote. However, feeding behavior components (feeding rate, searching rate, and handling time) are influenced by factors besides prey density, such as vigilance and flock size. In this study, we aim to elucidate how Bewick’s swans (*Cygnus columbianus bewickii*) adopt flexible foraging strategies and vary their feeding behavior components in response to disturbance, flock size, and food density. We collected focal sampling data on the foraging behavior of swans that foraged rice grains, foxnut seeds, and tubers in paddy field, foxnut pond, and lake habitats, respectively, in Shengjin and Huangpi lakes during winter from 2016 to 2018. The observed feeding rate was not correlated with food density and displayed a positive relationship with searching rate but negative relationships with handling time, flock size, overall vigilance time, and disturbance time. Handling time was negatively correlated with food density and flock size, yet it increased with disturbance, overall vigilance time, and normal vigilance time. Searching rate was negatively correlated with food density, flock size, and disturbance time. Feeding rate was affected by the combined effects of handling time and searching rate, as well as food density and searching rate. The shape of the observed functional response could not be fitted to Holling’s disc equation. However, the disc equation of the predicted feeding rate of wintering swans was found to be driven by food density. This provides insight into how wintering waterbirds adopt appropriate foraging strategies in response to complicated environmental factors, which has implications for wildlife conservation and habitat management.

## 1. Introduction

Understanding the changes in feeding rate in response to a variety of environmental conditions is a necessary tool to comprehend relationships between predators and prey, and tradeoffs among foraging strategies in animals [1]. Holling’s disc equation is the most commonly applied model [2,3]. This is a simple prey-dependent model that describes how the feeding rate of predators increases with prey density. It assumes that searching rate affects feeding rate when prey is difficult to find owing to low density and that handling time is more restricted as food density increases [4], i.e., that feeding rate becomes more heavily influenced by handling time as prey becomes easier to find. The Holling Type II functional response shows feeding rate in a decelerating rise to an asymptote at higher prey densities [5]. In a sense, waterbirds can be considered predators and their food items the prey. Therefore, a predator–prey relationship fitting a Type II functional response can be used to represent waterbirds and their food items [6]. Besides prey density, feeding behavioral components such as searching rate and handling time are influenced by other environmental factors in the surrounding habitat [7], especially by disturbance. Vigilance is an anti-predator behavior related to disturbance, and some studies show that it should be included in foraging behavior [8]. Many of these studies propose that feeding rate is a functional response to food density depending on estimates of search time, handling time, and vigilance time, or that there is a tradeoff between foraging effort and vigilance [9,10].

The components used to predict feeding rate as a functional response are interrelated. The patchiness of food (food density), group size of aggregated predators [11], and vigilance of the predators can affect the functional response, handling time [12], and searching rate of the predator [13]. Food patchiness represents the variation in food density among foraging habitats. For waterbirds, food density is associated with food abundance, and food availability is influenced by disturbance (conspecific individuals and interspecific predators). The free distribution theory reveals that the flock size of a predator changes with food density and availability [14,15]. The feeding rates of waterbirds can be constrained by flock size [2,16,17,18], and this is likely to happen in patches where food is highly aggregated [19,20]. Meanwhile, vigilance time also continually changes with the flock size of the predator [21]. An optimal forager adjusts its vigilance and foraging time to maximize fitness in risky habitats [22]. In addition, vigilance is reduced in patches with higher food density [23].

Beauchamp describes two types of vigilance, towards competitors and towards predators, and suggests that they have either no relationship or a negative relationship to food density [21]. Normal vigilance focuses on conspecific competitors and other avian herbivores such as geese and ducks that often feed in mixed flocks with swans [24,25]. Induced vigilance focuses on disturbance (e.g., predators) and has a significantly higher effect on feeding rate [26]. In addition, there seems to be a mixed relationship between functional response components (handling time and searching rate) and vigilance. The influence of vigilance on feeding rate is determined by the extent of overlap between vigilance and compatible handling time, which results from handling time constraining the feeding rate when predators forage in areas with high prey densities [10]. Searching rate is a function of food density [27], therefore, the effect of vigilance on feeding rate has a negative relationship with food density at a constant compatible handling time [28]. Studies on many species have focused on whether feeding rate varies with food density over a range of food densities and whether this conforms with Holling Type II functional responses [10,29]. Some studies showed results that did not conform with the predicted response [30,31,32]. However, these studies may not have considered the effect of vigilance on foraging behavior components. Smart *et al.* [10] constructed functional response models that describe how the time spent on vigilance limits handling and search time, and consequently affects feeding rate. For wildlife conservation and habitat management, it is important to understand how wintering waterbirds adopt appropriate foraging strategies and maximum fitness in response to complicated environmental factors that affect their feeding rates [33].

Some waterbirds, including Bewick’s swans (*Cygnus columbianus bewickii*) must obtain a large amount of food from their habitats to meet their necessary energy requirement [33,34], especially in wintering grounds. Swans usually prefer to feed on the underground storage organs (tubers) of certain aquatic plants [35], but these plants have declined and even disappeared under pressure from purse seine fisheries, increased water levels, and pollution stress at some key swan wintering sites [36,37]. Paddy fields and foxnut (*Euryale ferox*) ponds around lakes, where local farmers grow foxnuts, have become substitute foraging habitats. Throughout the foraging area, the majority of foraging sites have poor food availability for swans, as a result of habitat desiccation, and the swans are concentrated in only a few places with abundant food to forage (unpublished data). The characteristics of the foods available to the swans in paddy fields and foxnut ponds are different from those in the shallow lakes in the middle and lower Yangtze River floodplain. Swans feed on foxnut seeds and rice grains in foxnut ponds and paddy fields, respectively. Compared to the shallow lakes that provide tubers to wintering swans, these two habitats are more disturbed by human beings due to pedestrians and electric bicycles passing by. The foraging behavior responses of swans might vary according to the many complex interactions between human disturbance and food biomass.

We observed the foraging behavior of swans under different food density conditions in foxnut ponds, lake, and paddy field habitats that were situated close to roads and suffered from unintentional human disturbance. The aim of this study is to understand how swans adopt flexible foraging strategies (i.e., feeding rate, handling time, searching rate) and to use the disc equation to model how their responses to disturbance and food density vary. To achieve this, we measured the functional responses and associated behavioral parameters (i.e., handling time, searching rate, the proportion of time spent on overall vigilance, induced vigilance, and normal vigilance) of wintering swans. Based on previous studies, we test the following hypotheses: (a) the feeding rate and handling time, rather than the searching rate, increase with food density; (b) the feeding rate and handling time decrease while the searching rate increases as the number of individuals increases, owing to foraging competition; and (c) the feeding rate and searching rate decrease simultaneously while the handling time increases as more disturbance occurs. We also test whether the Type II Holling model and Smart models can predict the functional response of both observed and predicted feeding rates. We compare the predicted accuracy of these models and explore whether the functional response is primarily driven by food density or vigilance.

## 2. Methods

### 2.1. Study Sites

**Figure 1:**
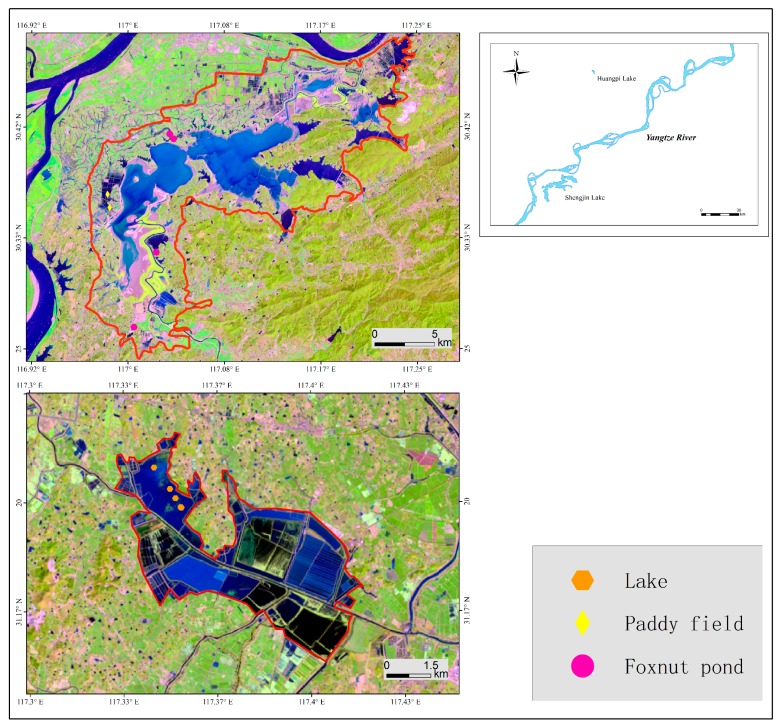
The location of foraging habitats for the wintering swans in Shengjin and Huangpi Lakes, in the middle and lower Yangtze River floodplain, Anhui Province, China. The green diamonds, pink dots, and white hexagons indicate the paddy field, foxnut pond, and lake study sites, respectively.

The studies were carried out in two lakes in the middle and lower Yangtze River floodplain, respectively: Shengjin Lake (30.27°~30.47°N, 116.98°~117°21′E) and Huangpi Lake (31.09°~31.12°N, 117.20°~117.24°E), Anhui, China (Figure 1). These lakes are traditionally important wintering refuges for waterbirds on the East Asian–Australasian Flyway [38]. Swans aggregate in lakes with relatively rich food resources, such as Shengjin Lake, and Huangpi Lake, as well as Caizi Lake and Baidang Lake [36]. They arrive in late October and migrate to their breeding grounds until the subsequent March.

The hydrology and vegetation of these lakes show marked changes during the over-wintering stage of the migrating waterbirds. In October water depth typically declines, vegetation undergoes senescence, and seeds ripen. By mid-winter, water depth has reached a minimum, vegetation has completed its senescence, and seeds have become dormant. From this point until the following March, water depth increases, whilst vegetation and seeds undergo germination [39].

At Shengjin Lake, the lake is degenerated as a result of reclamation and cultivation, and parts of the lake have been transformed into paddy fields and foxnut ponds at the lake shore. The submerged macrophytes that provide food for wintering waterbirds were destroyed by fishing, increased water levels, and pollution [37], which has impacted many species, including Bewick’s swans, which forage tubers. Swans also feed on the scattered seeds of rice (*Oryza sativa*) and foxnuts in the paddy fields and foxnut ponds as substitutional foraging habitats. Some rice and foxnut seeds are submerged after harvesting and are carried by water and covered with soil, resulting in variation in their quantities and densities. Compared with Shengjin Lake, Huangpi Lake is a small lake completely in the state of an artificial fish pond. *Vallisneria* spp. has been planted here as a food source for fish, and the tubers growing underground are utilized as a food resource by swans. Swans inhabit the fishing pond at Huangpi Lake during the winter.

Swans are routinely disturbed by fishing boats and farmers that appear at and next to the lake, respectively. Local residents are familiar with wintering swans, and there have been no incidents of harming swans. However, the activities of swans may have been affected by local people who live in proximity to their foraging areas, through activities such as walking and bicycling. Hence, walkers and electric bicycles may unintentionally disturb the normal foraging and vigilance activities of the swans.

The combination of food availability and human activities may encourage swans to aggregate. The flock size of swans changed vastly across all habitats, for instance, in foxnut ponds flock size ranged from 10 to more than 200 individuals. All swans observed in this study, nearly 180 individuals, migrated to paddy fields when foxnut ponds were not available. The number of swans foraging *Vallisneria* spp. fluctuated from almost a dozen to a hundred at Huangpi Lake (Supplementary Data).

### 2.2. Behaviors

Behavioral studies were conducted from late October to late March 2016–2018. We randomly selected an individual and used focal sampling [40] to record its behavior for about 10 min using a videotelescope (Victory PhotoScope 85 T*FL, 15-45X, Jena, Germany) after recording flock size and habitat type (paddy field, lake, or foxnut pond). The recorded behaviors were classified as vigilance or foraging. The overall vigilance behavior(v) included normal vigilance (v_n_, measured as a bending neck and watching mates’ positions) and induced vigilance (v_i_, measured as a head-up position with an erect neck, looking around the environment) [21]. Foraging behavior was classified into feeding, handling (handling was measured as mandibular movements to masticate a food item), and searching (head and/or body tilted down and submerged below the water’s surface). Feeding rate (i.e., number of prey consumed per unit time of active foraging), handling time (H, the time taken to masticate a food item), the proportion of time occupied by normal and induced vigilance (the ratio of time spent on different vigilance types and per video recording) were recorded. We derived estimates of the searching rate (a, m^2^ s^−1^) from the data of harvest rate against food density by fitting a Type II Holling disc equation [41]. We recorded 130, 51, and 49 video samples in the foxnut pond, lake, and paddy field habitats, respectively.

### 2.3. Disturbance

Most disturbances occurred due to local people passing by foraging sites close to roads and residences, i.e., walkers and cyclists. Because electric bicycles are small, we considered walkers and cyclists as creating the same level of disturbance. We recorded only the time duration of walkers and cyclists seen by swans in the foraging sites.

### 2.4. Food Item Density

We placed 3 quadrats (10 m × 10 m) in the lake at Huangpi Lake and 4 quadrats in paddy fields at Shengjin Lake at 100 m intervals in 2016/2017 and dug 8–12 sediment cores (11 cm diameter and 30 cm depth) in each quadrat. We placed 7 quadrats (150 m × 150 m, 6 at Shengjin Lake and 1 at Huangpi Lake) in three habitats, i.e., 1, 4, and 2 quadrat(s) in the lake, foxnut ponds, and paddy fields, respectively, in 2017/2018. We dug 30 sediment cores (25 cm diameter and 30 cm depth) per quadrat. We washed the sediment cores by sifting in a 3 mm sieve to isolate swan food sources. Foods were dried for 48 h at 60 °C until they reached a constant weight, then the weights of dried foods were recorded. Food density was calculated from the total dried food weights divided by total sediment core areas in a quadrat. We estimated the food item density by biomass density divided by the averaged weight of a single seed or tuber.

### 2.5. Functional Response Models

Four functional response models were fitted with observed data using linear models (LMs). Model 1, the Holling disc equation [42], describes that handling and searching behavior are mutually exclusive. The Model 2 adds “overall vigilance” based on Model 1 and assumes that overall vigilance, handling, and searching are mutually exclusive. Due to overall vigilance including both normal and induced vigilance, we subdivided Model 2 into Model 3 and Model 4. They assume that searching behavior and handling behavior are mutually exclusive; Model 3 includes only normal vigilance, and Model 4 contains only induced vigilance [10].

1. F = aD/(1+aDH) Model 1: Searching behavior and handling behavior are mutually exclusive, no vigilance.
2. F = (1−v)aD/(1+aDH) Model 2: Searching, handling, and overall vigilance are mutually exclusive.
3. F = (1−v_i_)aD/(1+aDH) Model 3: Searching behavior and handling behavior are mutually exclusive, no induced vigilance.
4. F = (1−v_n_)aD/(1+aDH) Model 4: Searching behavior and handling behavior are mutually exclusive, no normal vigilance.
  F = feeding rate (food items s^−1^), D = food density (food items m^−2^), a = searching rate (m^2^ s^−1^), H = handling behavior (s food item^−1^), v_n_ and v_i_ = proportion of time spent on normal and induced vigilance.

### 2.6. Statistical Analyses

A PIPI Player(version 3.4.0, Ku6 Media Co., Ltd., Beijing, China) was used to display and analyze the videos with frame by frame viewing on the computer. Vigilance (normal vigilance and induce vigilance), searching rate, handling time, flock size, and feeding rate data all displayed non-normal distributions and homoscedasticity, so we compared these data among three habitats using non-parametric tests.

To obtain the combined effect of interactive variables on the feeding rate, we used a two-way Permutational Multivariate Analysis of Variance (PERMANOVA) in Past 3 (a software for scientific data analysis, version 3.26, University of Oslo). We divided the handling time into four class intervals (0–1, 1–2, 2–3, and 3–4 s per food item). Searching rate included three parts (0–0.0001, 0.0001–0.001, 0.001–0.01 m^2^ s^−1^). Overall vigilance time percentages were divided into a low section (0–50%) and a high section (50–100%). Food item density was separated into 0–50, 50–150, 150–300, 300–500, 500–1000, and 1000–1500 food items m^−2^.

Linear models (LMs) were used to analyze the relationships between response and explanatory variables. We log10 transformed handling time, square-root transformed feeding rate, and arcsin transformed searching rate and vigilance (normal vigilance and induced vigilance). The observed feeding rates and predicted feeding rates were chosen as the dependent variables in four models, respectively. To test the given functional response models, we selected the explanatory variables as parameters into specific models that they need. We insured there was no collinearity in all models that contained multiple explanatory variables (correlation coefficients were less than 0.6 in all cases) [43].

To assess these four models, we compared their differing abilities to describe and predict the observed functional response. Their descriptive ability was measured using non-linear regression to fit a model to the observed functional response. We used directly observed parameter values from the natural environment to assess the ability of a model to predict the functional response. Variations in goodness-of-fit between models for the observed data were compared using the Akaike information criterion (AIC) in R (3.4.2). A significance level of 0.05 (*p*) was used for all statistical tests, and results were stated as mean ± SD.

## 3. Results

### 3.1. Handling Time

The mean handling time was 1.496 ± 0.741 s per food item. The highest value for handling time was in the foxnut ponds (1.659 ± 0.856 s food item^−1^), followed by the lake (1.293 ± 0.534 s food item^−1^), and the lowest value was in the paddy fields (1.276 ± 0.433 s food item^−1^). Handling time in the lake and paddy fields was similar, while it was significantly different in the foxnut ponds (foxnut ponds and lake *Z* = −3.021, *p <* 0.05; foxnut ponds and paddy fields *Z* = −3.390, *p* < 0.05; lake and paddy fields *Z* = −0.093, *p* > 0.05).

**Figure 2:**
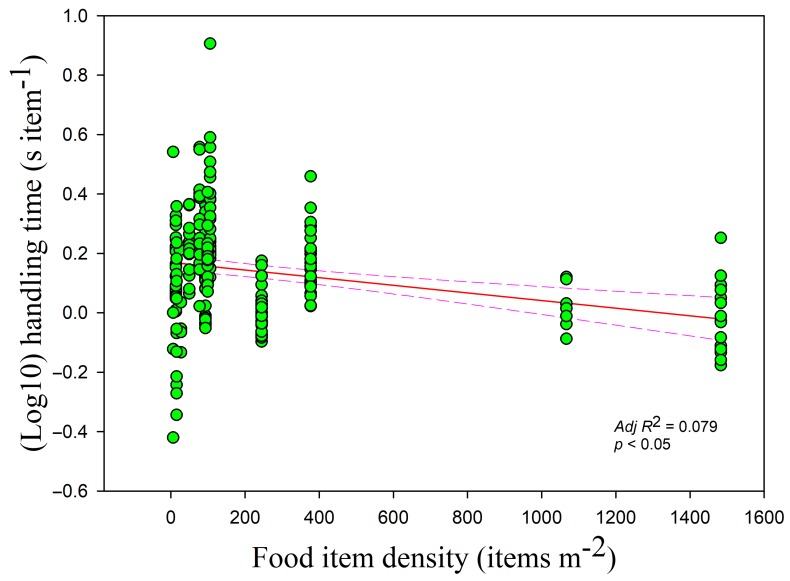
Handling time of swans in relation to food item density. Each point represents a single sample, and the regression line ± the 95% confidence interval are shown as solid and dashed trend lines, respectively.

**Figure 3:**
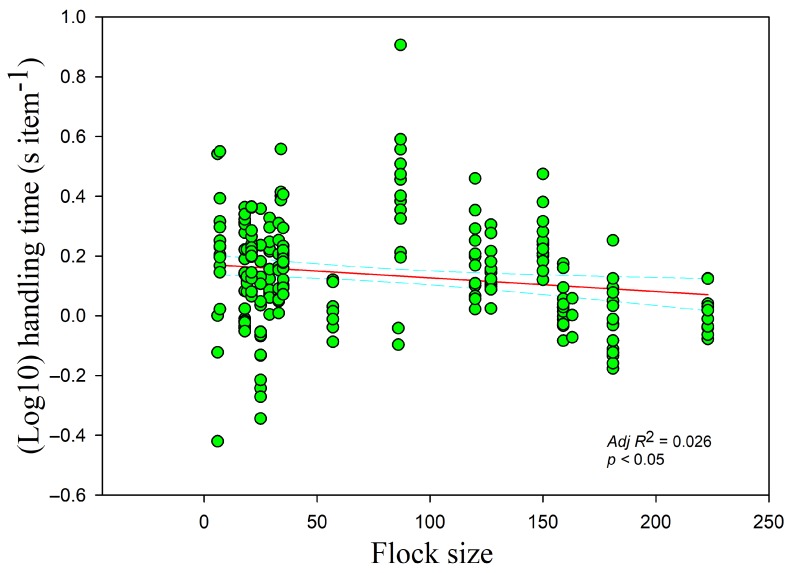
Handling time of swans in relation to flock size. Each point represents a single sample, and the regression line ± the 95% confidence interval are shown as solid and dashed trend lines, respectively.

**Figure 4:**
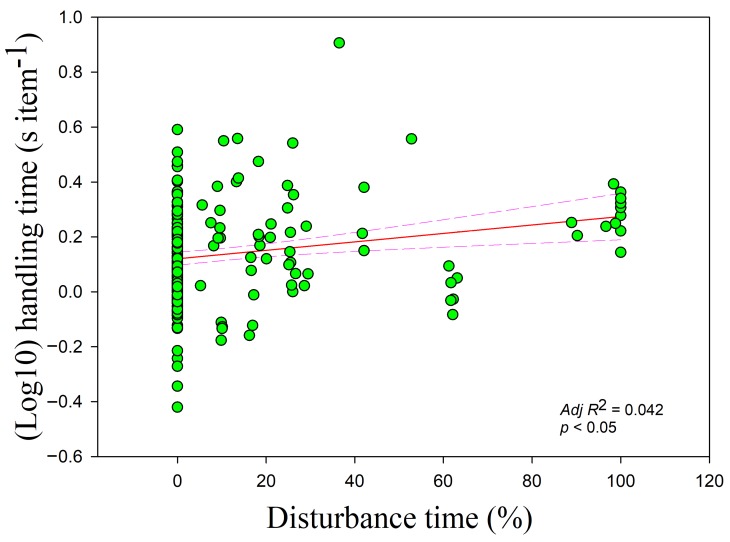
Handling time of swans in relation to disturbance time occurred. Each point represents a single sample, and the regression line ± the 95% confidence interval are shown as solid and dashed trend lines, respectively.

The handling time was negatively correlated with the food density overall (*r* = −0.233, *adj R*^2^ = 0.079, *F*_1,228_ = 20.559, *p <* 0.05) (Figure 2). Handling time was also negatively correlated with food density in the paddy fields and foxnut ponds, which had the highest and medium food densities, respectively (paddy fields *r* = −0.578, *adj R*^2^ = 0.401, *F*_1,47_ = 33.087; foxnut ponds *r* = −0.357, *adj R*^2^ = 0.254, *F*_1,128_ = 44.867; all *p* < 0.05) but showed no relationship in the lake, which had the lowest food density (*r* = −0.214, *p* > 0.05). The handling time had a negative relationship with the flock size (*r* = −0.140, *adj R*^2^ = 0.026, *F*_1,228_ = 7.107, *p* < 0.05) (Figure 3) but increased with disturbance time (*r* = 0.195, *adj R*^2^ = 0.042, *F*_1,228_= 10.955, *p* < 0.05) (Figure 4). The handling time was positively correlated with overall vigilance time (*r* = 0.199, *adj R*^2^ = 0.034, *F*_1,228_ = 9.063, *p* < 0.05), but upon closer examination, it was positively correlated with normal vigilance time (*r* = 0.285, *adj R*^2^ = 0.106, *F*_1,228_ = 28.099, *p* < 0.05) rather than induced vigilance time (*r* = 0.013, *p* > 0.05).

### 3.2. Vigilance

The mean percentages of disturbance time and flock size were 10.926 ± 24.310 % and 77.226 ± 66.347. They were the lowest in the lake (1.020 ± 5.100 % and 26.529 ± 6.906), followed by the foxnut ponds (12.334 ± 28.123 % and 76.177 ± 70.616), and the paddy fields (17.503 ± 22.650 % and 132.776 ± 40.814). Vigilance can be divided into two types: normal vigilance and induced vigilance. The time spent on the latter was higher than that spent on the former in the majority of samples. Flock size alone was significantly correlated with induced vigilance time (*r* = 0.158, *p* < 0.05) but not overall vigilance time or normal vigilance time (*r* = 0.098, *p* > 0.05; *r* = −0.031, *p* > 0.05, respectively). The percentage of time that the swans spent on overall vigilance was the highest in the paddy fields (9.153 ± 14.264 %), next highest was in the foxnut ponds (7.114 ± 10.680 %), and the lowest was in the lake (3.659 ± 5.672 %). The greatest values obtained for induced vigilance time and normal vigilance time occurred in the paddy fields (7.482 ± 14.264 %) and foxnut ponds (5.829 ± 8.821 %), respectively, while the lowest values occurred in the foxnut ponds (1.285 ± 4.528 %) and lake (0.773 ± 1.850 %), respectively.

The proportion of normal vigilance time differed among the three habitats. The ratios of overall vigilance time and induced vigilance time were significantly different between the habitats. However, no significant difference in the overall vigilance time ratio was observed between the lake and the foxnut ponds (*Z* = −1.802, *p* > 0.05). The proportions of time spent on overall vigilance and normal vigilance were negatively correlated with food density in the foxnut ponds, but that spent on induced vigilance was not (overall vigilance *r* = −0.227, normal vigilance *r* = −0.196, all *p* < 0.05, induced vigilance *r* = −0.149, *p* > 0.05). There were also no correlations between these vigilance time ratios and food density in the lake (overall vigilance *r* = −0.200, induced vigilance *r* = −0.234, normal vigilance *r* = 0.068, all *p* > 0.05). However, induced vigilance time showed a positive correlation with food density in the paddy fields (induced vigilance *r* = 0.324, *p* < 0.05), while normal vigilance time and overall vigilance time showed a negative correlation and no relationship, respectively (normal vigilance *r* = −0.297, *p* < 0.05; overall vigilance *r* = 0.272, *p* > 0.05).

The disturbance time, overall vigilance time, and induced vigilance time were all positively correlated with food density (disturbance ratio *r* = 0.233; overall vigilance *r* = 0.179; induced vigilance *r* = 0.364; all *p* < 0.05), unlike the normal vigilance time (*r* = −0.143, *p* < 0.05). Overall vigilance time and normal vigilance time were not correlated with flock size, unlike induced vigilance time (overall vigilance *r* = 0.098, normal vigilance *r* = −0.031, all *p* > 0.05; induced vigilance *r* = 0.158, *p* < 0.05). Overall vigilance time was positively correlated with disturbance time (*r* = 0.596, *adj R*^2^ = 0.353, *F*_1,228_ = 125.696, *p* < 0.05), as were normal vigilance time (*r* = 0.463, *adj R*^2^ = 0.213, *F*_1,228_= 63.040, *p* < 0.05) and induced vigilance time (*r* = 0.386, *adj R*^2^ = 0.145, *F*_1,228_ = 39.801, *p* < 0.05).

### 3.3. Searching Rate

The mean searching rate was 0.007356 ± 0.013312 m^2^ s^−1^.The searching rate was highest in the lake (0.029195 ± 0.01326 m^2^ s^−1^), followed by the paddy fields (0.002944 ± 0.002495 m^2^ s^−1^), and the foxnut ponds (0.000451 ± 0.000301 m^2^ s^−1^). Significant differences were observed among the three habitats (lake and foxnut ponds *Z* = −10.454, foxnut ponds and paddy fields *Z* = −9.330, lake and paddy fields *Z* = −8.581, all *p* < 0.05).

**Figure 5:**
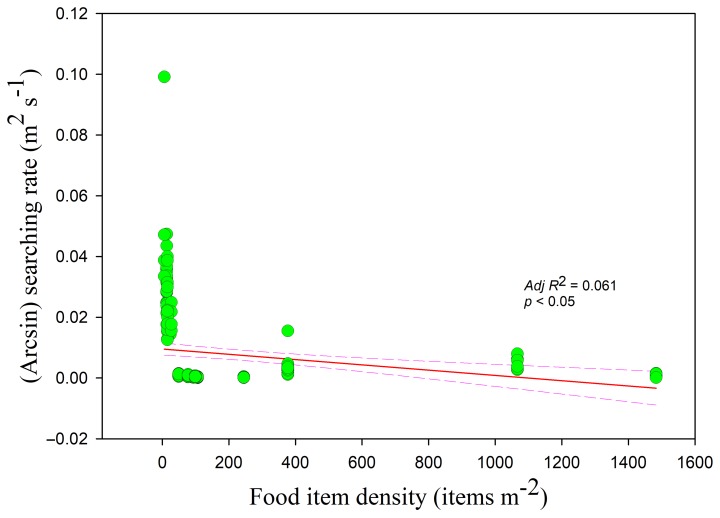
Searching rate in relation to food item density. Each point represents a single sample, and the regression line ± the 95% confidence interval are shown as solid and dashed trend lines, respectively.

**Figure 6:**
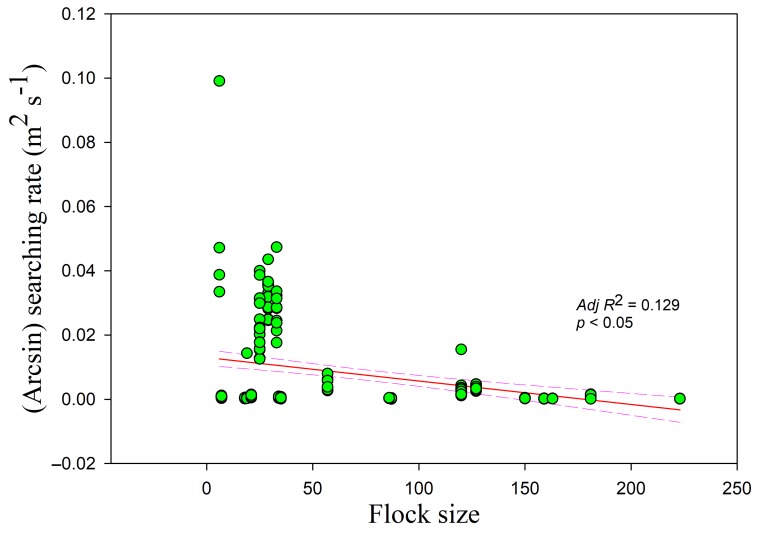
Searching rate in relation to flock size. Each point represents a single sample, and the regression line ± the 95% confidence interval are shown as solid and dashed trend lines, respectively.

**Figure 7:**
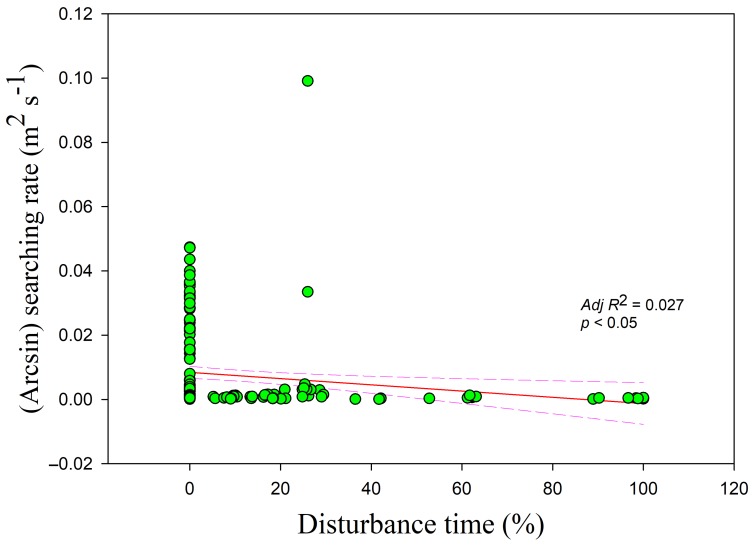
Searching rate in relation to percentage disturbance time occurred. Each point represents a single sample, and the regression line ± the 95% confidence interval are shown as solid and dashed trend lines, respectively.

The searching rates in all three habitats showed negative correlations with food density (*r* = −0.255, *adj R*^2^ = 0.061, *F*_1,228_ = 15.807; lake *adj R*^2^ = 0.250, *F*_1,49_ = 17.644; foxnut ponds *adj R*^2^ = 0.353, *F*_1,128_ = 71.396; paddy fields *adj R*^2^ = 0.140, *F*_1,47_ = 8.812, all *p* < 0.05) (Figure 5). The intercept was highest and lowest in the paddy fields and foxnut ponds, respectively. The searching rate was also negatively correlated with flock size (*r =* −0.364, *adj R*^2^ = 0.129, *F*_1,228_ = 34.774, *p* < 0.05) (Figure 6) and disturbance time (*r =* −0.178, *adj R*^2^ = 0.027, *F*_1,228_ = 7.364, *p* < 0.05) (Figure 7).

### 3.4. Feeding Rate

The mean observed feeding rate was 0.017 ± 0.007 food items s^−1^. The observed feeding rate was lowest in the paddy fields (0.015 ± 0.006 food items s^−1^), followed by the foxnut ponds (0.017 ± 0.006 food items s^−1^) and the lake (0.020 ± 0.008 food items s^−1^). The observed feeding rate showed no significant difference between the lake and the foxnut ponds (*Z* = −1.690, *p* > 0.05) but was significantly different between the paddy fields and the other habitats (lake and paddy fields *Z* = −2.927, foxnut ponds and paddy fields *Z* = −2.255, all *p* < 0.05).

**Figure 8:**
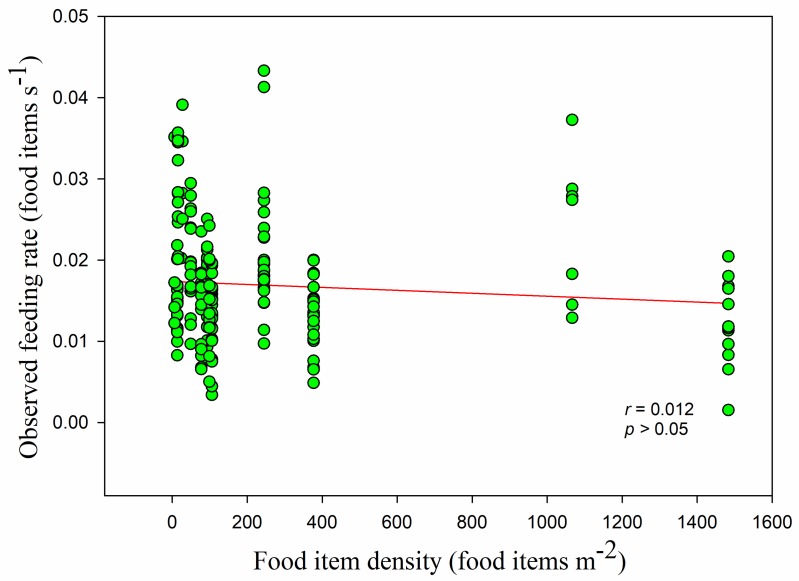
The observed feeding rate in linear relation to food item density. Each point represents a single sample, and the linear relation is shown as a red line.

**Figure 9:**
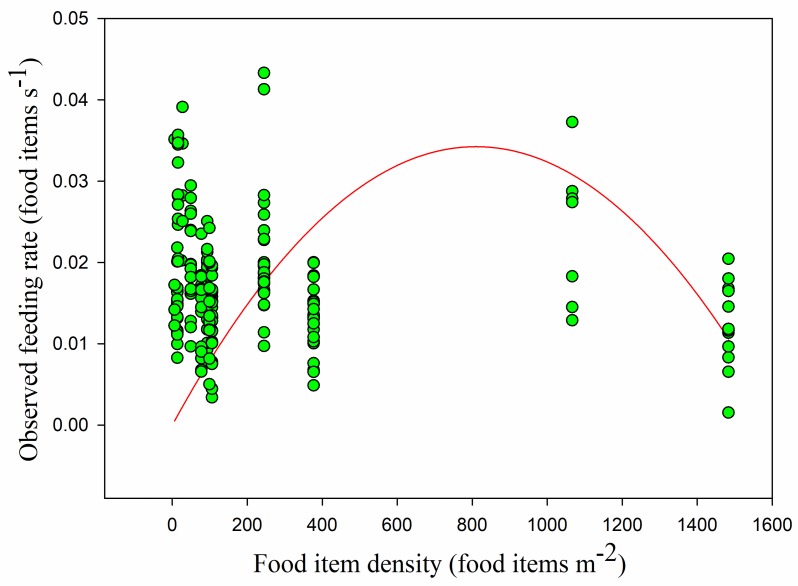
The observed feeding rate in curve relation to food item density. Each point represents a single sample, and their relationship is shown as a curved line.

The mean food density was 251.91 ± 391.00 food items m^−2^. The food density in the paddy fields (837.99 ± 511.93 food items m^−2^) was higher than that in the foxnut ponds (124.04 ± 68.45 food items m^−2^) and lake (14.78 ± 4.59 food items m^−2^). The quantity of food items in the paddy fields was nearly ten times that in the lake, and there were significant differences between all three habitats (lake and foxnut ponds *Z* = −10.545, lake and paddy fields *Z* = −8.824, foxnut ponds and paddy fields *Z* = −10.394, all *p* < 0.05). Overall, the observed feeding rate had no linear relationship with food density (*r* = −0.102, *p* > 0.05) (Figure 8). This was also the case in the paddy fields, but feeding rate was significantly positively correlated with food density in the foxnut ponds and lake (foxnut ponds *r* = 0.284, *adj R*^2^ = 0.068, *F*_1,128_ = 10.399, *p <* 0.05; lake *r* = 0.447, *adj R*^2^ = 0.184, *F*_1,49_ = 12.248, *p <* 0.05; paddy fields *r* = 0.092, *p >* 0.05). However, the disc equation could not be used to verify the observed relationship between food density and feeding rate (non-linear regression, *adj R*^2^ = 0.015, *F*_1,228_ = 2.752, *p* > 0.05) (Figure 9). 

**Figure 10:**
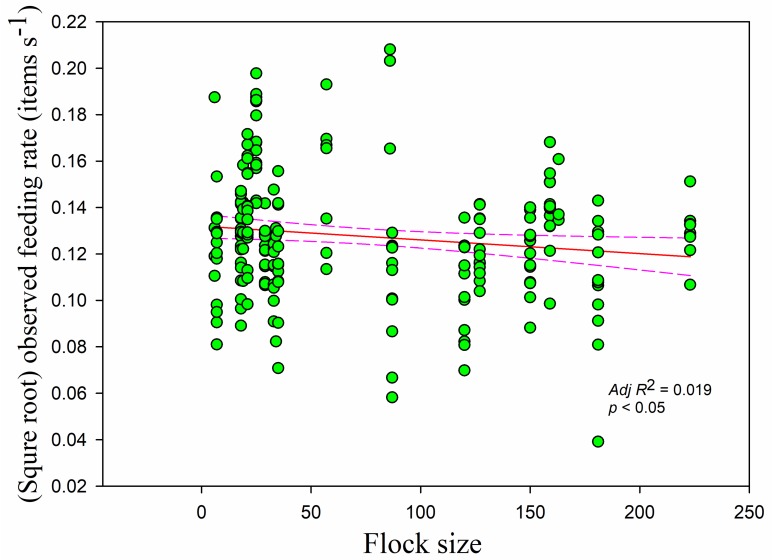
The observed feeding rate in relation to flock size. Each point represents a single sample, and the regression line ± the 95% confidence interval are shown as solid and dashed trend lines, respectively.

**Figure 11:**
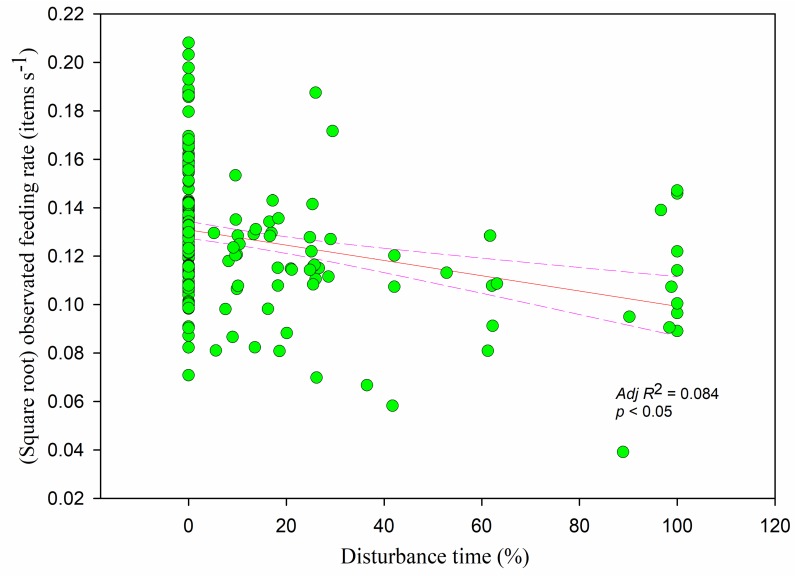
The observed feeding rate in relation to disturbance time. Each point represents a single sample, and the regression line ± the 95% confidence interval are shown as solid and dashed trend lines, respectively.

The observed feeding rate was negatively correlated with flock size (*r* = −0.156, *adj R*^2^ = 0.019, *F*_1,228_ = 5.335, *p <* 0.05) (Figure 10) and decreased significantly with increasing overall vigilance time (*r* = −0.380, *adj R*^2^ = 0.198, *F*_1,228_ = 57.421, *p* < 0.05) and disturbance time (*r* = −0.259, *adj R*^2^ = 0.084, *F*_1,228_ = 21.923, *p* < 0.05) (Figure 11). The feeding rate decreased as handling time increased (*r* = −0.283, *p* < 0.05), showing a linear relationship (*adj R*^2^ = 0.096, *F*_1,228_= 25.187, *p* < 0.05). Feeding rate and searching rate showed a positive relationship (*r* = 0.253, *adj R*^2^ = 0.054, *F*_1,228_ = 13.946, *p* < 0.05).

**Figure 12:**
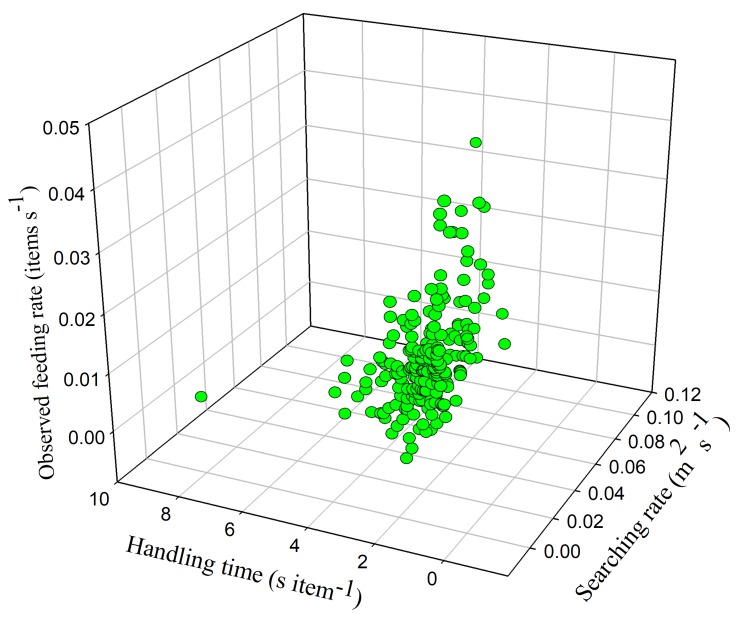
Swans’ feeding rate in relation to searching rate and handling time.

**Figure 13:**
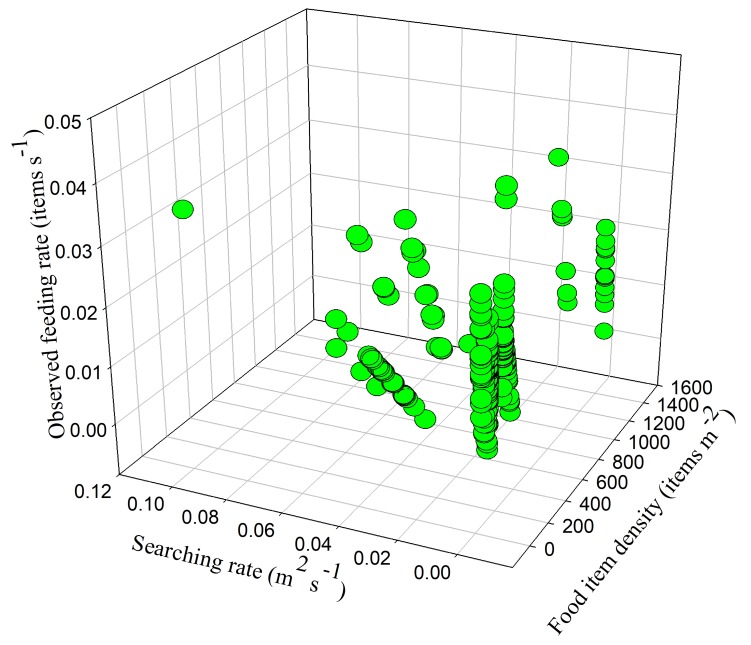
Swans’ feeding rate in relation to searching rate and food density.

Handling time and searching rate had a combined effect on feeding rate (*F*_6,218_ = −16.332, *p* < 0.05) (Figure 12), which means that the swans invested more effort in searching and reduced their food handling time to increase their feeding rate. Food density and searching rate also had an interactive effect on feeding rate (*F*_26,188_ = −4.432, *p* < 0.05) (Figure 13), which shows that feeding rate increased with searching rate in foraging sites that had lower food densities. However, the combined effect of overall vigilance time and food density on feeding rate was insignificant (*F*_13,202_ = −9.582, *p* > 0.05), as were the combined effects of flock size and overall vigilance time (*F*_19,190_ = −6.151, *p* > 0.05), handling time and overall vigilance time (*F*_3,222_ = −52.287, *p* > 0.05), and handling time and food density (*F*_39,174_ = −2.971, *p* > 0.05). This shows that the swans did not adapt their feeding rate in response to the interactive effects of food density, flock size, and vigilance.

### 3.5. Comparing Fitted and Predicted Functional Responses

We obtained four predictive functional response models using observed data (handling time, overall vigilance time, normal vigilance time, and induced vigilance time) and a derived searching rate in order to identify which combination of parameters produced the most accurate predictions.

Models 1–4 were parameterized using the observed behavioral parameters mentioned above, and their predictions (observed feeding rates) for the functional responses were compared. Model 1, Holling’s disc equation, had the lowest AIC (−505.933). Model 2, Model 3, and Model 4 obtained the same AIC for the observed feeding rate (−233.240), and it was larger than that obtained for Model 1 (Table 1). Consequently, we concluded that Model 1 provided the best fit for the observed data even if the observed feeding rate was not consistent with the Holling Type II functional response (Figure 9).

**Figure 14:**
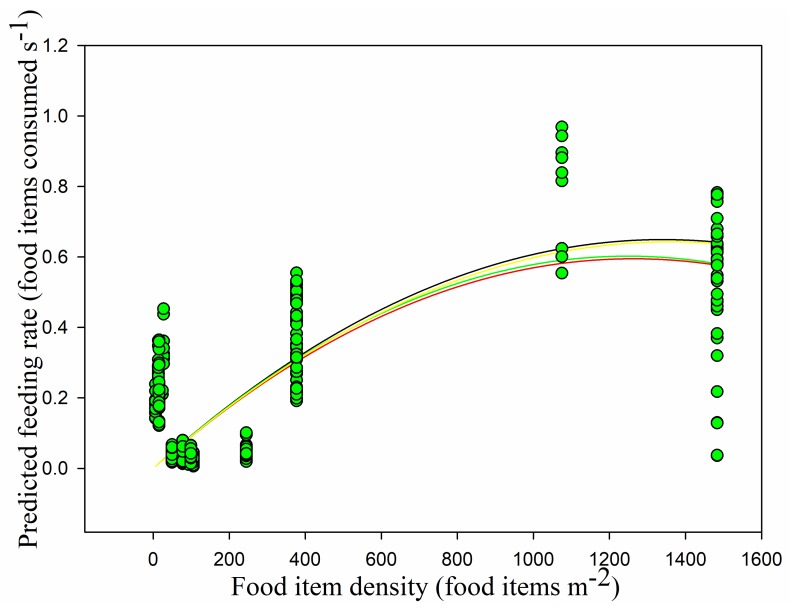
The relationship between food item density and predicted feeding rate under various models. Model 1, which predicts feeding rate using Holling’s disc equation, is indicated by the black line. Model 2, which predicts feeding rate using the Smart model with overall vigilance, is indicated by the red line. Model 3, which predicts feeding rate using the Smart model with induced vigilance only, is indicated by the green line. Model 4, which predicts feeding rate using the Smart model with normal vigilance only is indicated by the yellow line.

For the predicted functional responses, Holling’s disc equation had the lowest AIC (−420.883) and the best goodness-of-fit (*adjR*^2^ = 0.511, *F*_1,228_ = 240.225, *p* < 0.05). Model 2 obtained a higher AIC for the predicted feeding rate (−82.200) and a lower goodness-of-fit (0.450). The AIC of Model 3 was lower than those of Model 2 and Model 4, and its goodness-of-fit was the same as that of Model 2 (0.450). The predicted feeding rate in Model 4 had the highest AIC (−64.564) and the second highest *R*^2^ (0.510). We concluded that Model 1 was the most accurate predictive model when using only handling time and searching rate (Table 1, Figure 14).

## 4. Discussion

In this study, we identified that food density, vigilance, and flock size influence the foraging behavior of a flagship wintering waterbird, especially foraging elements such as the feeding rate, searching rate, and handling time. The first and second hypotheses, namely, (a) the feeding rate and handling time, rather than the searching rate, increase with food density, and (b) the feeding rate and handling time decrease as the number of individuals increases, while the searching rate increases, were partially affirmed. However, only the third hypothesis, (c) the feeding rate and searching rate decrease simultaneously while the handling time increases with increase in disturbance, was validated. Moreover, the observed feeding rate could not be predicted from the vigilance time, handling time, derived searching rate, and food density using the disc equation. However, the predicted feeding rate in Holling’s functional response model was primarily driven by food density.

We found that handling time was reduced significantly at higher food item densities. Mandibular movements have an estimated duration of approximately 1.5 s, and the values in this study were lower than those in a study by Nolet *et al*. [44], which was conducted in creeks with abundant food. A possible reason that swans spend less time manipulating individual food items at high food densities is that there is an abundant alternative food supply. When there is a low availability of food, swans may spend more time handling each food item, breaking it down to increase digestibility and maximize energy gain. This is less necessary when food is present at high density [10]. In addition, greater flock size means more individuals competing for food. This could be another reason that swans decrease handling time to search for the next food item [29].

Animals can be alert to disturbance as they handle food [29]. For swans, predators that can simultaneously handle food and be alert to the environment or look for mates, handling behavior serves as an indispensable tool that allows them to adapt to increasing disturbance. In our study, the handling time decreased with increasing food density and greater flock size in the foxnut ponds and paddy fields, which are close to villages and thus have a high level of human disturbance, indicating that the swans inevitably face unintentional human effects. However, there was no relationship with food density in the lake that had lower disturbance, fewer food items, and attracted smaller flocks. A possible reason for this could be that swans spend more time observing the environment and other individuals in the flock while they are handling food [21,29], and increased disturbance time directly increases handling time [10].

Our results show that normal vigilance, rather than induced vigilance, accompanied the handling of food. Normal vigilance could probably be used to observe the foraging and vigilance behavior of intraspecific individuals [21]. Semipalmated sandpipers (*Calidris pusilla*) increase normal vigilance to monitor their neighbors when individuals are close together [45], and this has also been observed in other species [46]. We observed the same behavior in paddy fields and foxnut ponds with larger flocks. The swans reduced their normal vigilance time when food density decreased in the paddy fields and foxnut ponds were selected by more foraging swans, and this was likely the result of foraging competition and foraging pressure. The wintering swans faced flock concentration, food shortages, and conspecific competition. As such, they may perform more normal vigilance to observe conspecifics searching for food, in order to gain a competitive advantage. On the other hand, they may understand the environmental conditions by observing companions. As the normal vigilance time was less than the induced vigilance time, swans spent more time observing conspecifics, resulting in the better prediction ability of Model 4 compared to Models 2 and 3. In addition, the effect of normal vigilance on foraging behavior resulted in the Type II functional response predicting swan feeding behavior slightly better than Model 4.

The induced vigilance time reduced with flock size, which is in accordance with the “more eyes” theory. Induced vigilance behavior increased in response to higher food density and disturbance in the paddy fields, which is similar to the results of several studies on granivorous birds [10,47]. Forager group size was found to affect induced vigilance but not in predators foraging in high density food patches [21]. Baker [28] reported that grey partridges (*Perdix perdix*) devote more effort to being alert in circumstances with low food availability, which differs from our results. This phenomenon likely results from swans foraging in small patches that are too close to disturbance, leading to all the individuals needing to enter disturbed areas for food. In this study, we only focused on the time spent on vigilance caused by slight disturbances, such as passers-by and electric bicycles. However, the variety in disturbance type and magnitude, i.e., the source, distance, direction, and location of the disturbance, and the type of cover in the habitat, may result in variation in vigilance duration [22,48]. To develop a full and accurate picture of functional responses, additional studies are needed that investigate the factors likely to influence vigilance behavior.

Compared to the searching rates obtained by Nolet and Klaassen [3] from swans at a stopover habitat in Luareenmeer in the Netherlands (0.00102 and 0.000612 m^2^s^−1^), the values that we obtained from the paddy fields were only slightly higher. Further data collected in experiments by Nolet *et al*. (0.000201–0.000251 m^2^ s^−1^) were similar to our data from the foxnut ponds [49]. These were higher than the values from the paddy fields and lower than those from the lake. The possible reasons for this variation are differences in food abundance and the depth at which the food is buried underground. The primary food of Bewick’s swans foraging in creeks was pondweed (*Potamogeton pectinatus*) tubers that were buried below the ground [48,50]. However, the wintering swans that we studied fed on foxnuts and rice grains in artificial circumstances, as well as the pondweed tubers in lakes. The quantity of rice grains is higher and the size smaller, while the quantity of foxnuts is lower, but their size is 3–4 times greater. The density of tubers in the lake is the lowest of the three food sources investigated, the probability of a swan finding a tuber is lower each time. The abundance of foxnuts and rice grains was higher than that of the tubers, and as a result, the swans could find these resources more easily and invest less time in searching for them. The amount of time swans spent with their head underwater may not only represent search time as this time could also contain handling time. However, the probability of this is low as due to the large size of the tubers, it was difficult for swans to feed on tubers underwater. However, direct feeding on grains underwater may occur in paddy fields [51]. In addition, rice grains and foxnuts are dropped into the water and then buried by the flowing water and sediments, the depth to which these were buried was usually shallower than that of the tubers that grew underwater. It was also easier for the swans to probe for rice grains and foxnuts than for tubers. Searching rate reduced with flock size, which may be because more individuals can more easily find food [52,53].

Holling models indicate that feeding rates are increasingly constrained by handling time, rather than by searching rate, as food density increases [5]. Our study also shows that the feeding rate decreased with handling time, however, this result is different from that obtained for most wading birds [54], i.e., that there is no functional response between feeding rate and handling time in wading birds. A possible reason for this is that the swans were performing other activities, such as induced and normal vigilance, while handling their food. Feeding rate also changes with prey selection [55] and is influenced by food size. Reductions in the ingestion time of rice grains, foxnuts, and tubers are probably owing to their size, which has been reported in greylag geese (*Anser anser*) consuming *Scirpus maritimus* tubers [56]. 

The functional responses of the observed feeding rates of wintering swans did not fit the disc equation based on the observed handling time and derived searching rate. This phenomenon was similar to reports for a range of wading birds that feed on macro-invertebrates [7]. Although the feeding rates of some wading birds had Type II functional responses, the observed feeding rates were far lower than the predicted functional responses estimated by the disc equation using handling time and searching rate, i.e., the disc equation greatly overestimated the feeding rate. Some factors that affected foraging could perhaps explain these differences, for example, prey crypticity [57] and disturbance. The prey crypticity of food of the swan encompasses the complex variations in searching rate according to different habitats and food types. It is easier to search for a large foxnut than a small rice grain when they are available en masse. In addition, these foods are also not buried as deeply as tubers. At lower food densities, the swans searched larger areas over the same amount of time than they did at higher food densities. In addition, Blanchard and Fritz [26] highlighted that normal and induced vigilance have different disturbance effects on waterbirds and that the latter has a significant effect on feeding rate. Disturbance increased handling time, resulting in the increase of the denominator of the disc equation and higher prediction values than those in models that did not include interference. Consequently, the feeding rate was influenced indirectly and was lower than that predicted by the model. Feeding rate decreased with disturbance time and increased with food density in the lake and foxnut ponds, but not in the paddy fields. This results from paddy fields with higher disturbance leading to swans requiring more handling time and vigilance to observe their surroundings. In addition, the feeding rate was lower for rice grains due to swans feeding underwater in the paddy fields [51], which resulted in some feeding behaviors hardly being observed and a decreased feeding rate at maximum item density. However, since the sizes of both tubers and foxnut seeds are large, it was estimated that swans fed on only one food item each time and fed above water.

Although the feeding rates obtained in all three habitats were lower than those of swans in Europe, our calculated feeding rates in the lake were similar to those of Anatidae foraging for tubers at Shengjin Lake and those of wintering greylag geese [56]. A likely explanation is that both food density and abundance are lower in the degraded habitats than in the original habitat. Studies have shown that the functional responses and behavior of waterbirds are the most strongly influenced by the substrate, with variations in substrate type and compaction affecting search and feeding rates [58]. Stubble height can also affect feeding rate [28]. Therefore, consistency in food type and habitat and a lack of disturbance should be taken into account in future studies to satisfactorily measure the most appropriate functional response model.

The observed feeding rate was constrained by flock size, which was similar to the findings of previous studies on foraging waterbirds [2,16,18]. Decreases in feeding rate were attributed to inevitable interference, such as conflicts and avoidance movements [17]. Although the observed feeding rate was negatively related to flock size, food density, and vigilance, the model that predicted feeding rate most accurately included food density rather than flock size or vigilance time. Flock size is likely to become increasingly important as aggregated food attracts more wintering waterbirds in periods of short food supply, however, the food supply in this study seemed sufficient to supply the swans non-aggressively.

## 5. Conclusions

This study confirms that wintering swans have flexible foraging strategies that they use under different conditions in heterogenetic habitats. The observed feeding rate was not correlated with food density and had a positive relationship with searching rate, however, it was negatively correlated with handling time, flock size, vigilance time, and disturbance time. Handling time was negatively correlated with food density and flock size, yet it increased with disturbance and vigilance time (normal vigilance time rather than induced vigilance time). Searching rate did not change with handling time, yet it was negatively correlated with food density, flock size, and disturbance time. The disc equation could not predict the shape of these functional responses and even overestimated the feeding rate. However, food density, rather than vigilance, affected the predicted functional responses of the wintering swans, and it was recognized that the disc equation for the feeding rate was still driven by food density. For wintering waterbirds foraging in a range of situations with changing resources, these findings may help us to understand the effect of food density on the functional responses of feeding rates and to develop appropriate policies for conservation management.

## Figures and Tables

**Table 1:** The Akaike information criterion (AIC) comparing the ability of Models 1–4 to fit the fitted and predicted functional response.

Items	Holling Model	Smart Model_vigilance_	Smart Model_induced vigilance_	Smart Model_normal vigilance_
	Model 1	Model 2	Model 3	Model 4
No. of parameters	6	2	2	2
AIC of fitted functional response models	−505.933	−233.240	−233.240	−233.240
*R*^2^ of fitted functional response models	-	-	-	-
AIC of predicted functional response models	−420.883	−82.200	−79.197	−64.564
*R*^2^ of predicted functional response models	0.511	0.450	0.450	0.510

Type II Holling model (Holling 1959b); Smart model (Simon L. Smart 2008); -, no relationship.

## Supplementary Materials

The following are available online at https://www.mdpi.com/2076-2615/9/11/946/s1, Table S1: Raw data of foraging behaviors and microhabitat factors of wintering swans.
